# Access to assistive technology in two Southern African countries

**DOI:** 10.1186/s12913-018-3605-9

**Published:** 2018-10-19

**Authors:** Rebecca A. Matter, Arne H. Eide

**Affiliations:** 1 Faculty of Health Sciences, School of Public Health and Family Medicine, University of Cape Town, Falmouth Rd., Observatory, Cape Town, 7925 South Africa; 2 SINTEF Technology and Society, Oslo, Norway; 3 Centre for Rehabilitation Studies, Stellenbosch University, Stellenbosch, South Africa

**Keywords:** Disability, Assistive technology, Self-help devices, Health access, Mobility, Southern Africa, Botswana, Swaziland, Low-income countries

## Abstract

**Background:**

Millions of people in Southern Africa are deprived of basic human rights such as the right to education and work because of the large and growing unmet demand for assistive technologies (AT). Evidence is needed to better characterize the lack of AT access.

**Methods:**

This study serves to identify the sociodemographic factors that are associated with access to AT in two countries in Southern Africa, Botswana and Swaziland. To achieve this aim, logistics regression was applied to a subset of variables from two Living Conditions Studies, nationally representative surveys that were conducted in Southern Africa (2014 and 2010).

**Results:**

In Botswana, 44% of people who needed AT did not receive it, while in Swaziland the unmet need was 67%. Among the sociodemographic variables tested, the type of disability was the most important factor in determining AT access in both countries. The likelihood of AT access was highest in both countries for those who had mobility limitations (i.e., difficulty walking/climbing stairs) [Botswana: 6.4 odds ratio (OR) = 6.4., 95% confidence internal (CI) (3.6–11.3); Swaziland: OR = 3.2, CI (1.4–7.3)], in comparison to those with non-mobility types of disabilities.

**Conclusions:**

These findings provide support for governments and other stakeholders in the AT sector to prioritize AT to address the large unmet demand, and expand the range of AT products provided so that people with hearing, seeing, self-care, communication and cognition difficulties have equal access to AT as those with mobility impairments. A step toward achieving these aims is to inventory AT product types that are commonly covered through the public sector in each country, and identify common gaps (e.g., daily living aids). Advancing the AT sector as a whole within Southern Africa will require large scale qualitative studies that achieve a comprehensive understanding of the bottlenecks in regional AT supply, procurement, and delivery systems.

**Electronic supplementary material:**

The online version of this article (10.1186/s12913-018-3605-9) contains supplementary material, which is available to authorized users.

## Background

Millions of people in Southern Africa are deprived of basic human rights such as the right to education and work because of the unmet demand for assistive technologies (AT) [1, 2]. The World Report on Disability uses the following definition of AT; “Any item, piece of equipment or product system, whether acquired commercially, off-the-shelf, modified or customized, that is used to increase, maintain, or improve functional capabilities of individuals with disabilities” [3]. A few examples of AT include prosthetics, hearing aids, spectacles, white canes and adaptive eating utensils. Increasing access to AT in Southern Africa requires more products and services – in terms of quantity, quality and variety – as well as the reduction of barriers to existing AT. To achieve these aims, a comprehensive understanding of demand and supply-side facilitators and barriers is critical. This study serves to identify the sociodemographic factors (demand-side) that are associated with AT access in two countries in Southern Africa, Botswana and Swaziland. This demand-side analysis of national survey data aims to increase our understanding of who is accessing and not accessing AT.

For this study, Southern Africa refers to the 15 countries that comprise the Southern African Development Community [4]. All member states in Southern Africa, with exception of Botswana, have signed the UN Convention on the Rights of Persons with Disabilities (CRPD), that explicitly addresses the provision of AT in numerous Articles (i.e., 4, 9, 20, 21, 24, 26, 29 and 32) [5]. Researchers have also shown that access to AT is critical to achieving all of the 17 Sustainable Development Goals [6]. Yet achieving these rights and goals are out of reach when the AT sector in Southern Africa continues to be under funded, fragmented, and not well understood.

Researchers have begun to develop an inventory of demand-side factors that may determine access to AT in Southern Africa, but have yet to prioritize these factors or determine their relationships to each other. Sociodemographic factors mentioned in a recent publication on AT provision and outcomes in four Sub-Saharan Africa countries (South Africa, Namibia, Malawi and Sudan) included age, gender, poverty, location (rural vs. urban), and type of disability [2]. Existing evidence on AT in Southern Africa continues to be dominated by studies on mobility and vision devices, with few studies on hearing or communication related AT, and virtually no research on cognitive AT [7]. These studies generally focus on one country and one type of AT so fail to provide evidence about the AT sector as a whole in the Southern Africa region.

In this study, we posited that rurality may be the most important factor in explaining AT access in Southern Africa as the majority of AT providers are in urban centers, and rural residents face numerous barriers to accessing health care and other services in Southern Africa [8–10]. Rural location has also been associated with lack of AT access in a few other Southern African studies [11–13]. An alternative theory is that disability type (i.e., mobility impairments) is the most important factor as mobility devices are the most commonly available type of AT in Southern Africa [2]. To test our hypotheses, we analyzed a subset of cases and sociodemographic variables within the Living Condition Studies for Botswana and Swaziland (Table 1) [14, 15]. Living Conditions Studies (LCS) are nationally representative surveys that capture a wide range of social and economic living conditions of people with disabilities. These surveys not only aim to measure economic and material status but also the degree to which people with disabilities participate in major life activities (i.e., education, employment, community) and realize their human rights, including the right to health care and AT [16]. According to the LCS reports, over half of the total populations of people with disabilities in Botswana (59.1%) and Swaziland (57.3%) reported that they needed AT [14, 15].

**Table 1:** Living Conditions Studies in Botswana and Swaziland

Country	Implementing partners	Data collection	Included Cases
Botswana	The Botswana Federation of the Disabled (BOFOD), Southern Africa Federation of the Disabled (SAFOD), University of Botswana, Statistics Botswana, Office of the President – Botswana, Norwegian Federation of Organisations of Disabled People (FFO), SINTEF.	2012–14	486
Swaziland	The Federation of Organizations of the Disabled in Swaziland (FODSWA), Central Statistical Office.	2009–10	332

As of December 2017, LCS have been carried out in 9 countries since 2004, primarily within Southern Africa, and offer the most comprehensive snapshot of AT access in Southern Africa to-date. Botswana and Swaziland were selected because the studies were completed most recently among the 9 LCS studies, permissions were obtained to conduct the secondary analysis, and sample size was adequate for performing logistics regression.

Cases selected for inclusion were individuals with disabilities 15 years of age or older who reported needing AT device services. We excluded data from persons under 15 years because the surveys did not include questions about employment about education - two factors examined in this study. Through applying logistics regression, we were able to identify the most important demand-side characteristics that explain AT access in each country. While some comparisons are made between the countries, separate logistics regression models were developed because each country has a distinct profile (Table 2), and data collection took place over 3 years apart.

**Table 2:** 2010 World Bank - World Development Indicators

	Botswana	Swaziland
Total population (millions)	2.0	1.2
Population density (people per sq. km of land area)	3.6	69.9
Poverty headcount ratio at national poverty lines (% of population)	19.3%	63.0%
GNI per capita, Atlas method (current US$)	$5570	$3070
Income share held by lowest 20%	2.8%	4.0%
Life expectancy at birth, total (years)	60	51

Source: https://data.worldbank.org/

As shown in Table 2, Botswana is sparsely populated and the gross national incomes (GNI) per capita is nearly double that of Swaziland. Swaziland is densely populated and reports 63% of the population living below the poverty line.

## Methods

### Living conditions study survey

The original questionnaire for the LCS was based on two instruments: 1) a national disability survey for South Africa [17], and 2) a study on living conditions of the general population in Namibia [18]. Revisions were then made to this questionnaire to ensure relevance within each country where it was implemented. SINTEF Technology and Society worked in partnership with FFO, SAFOD, national disability organizations, researchers and central statistical offices, to implement studies and included people with disabilities as supervisor and research assistants.

The target sampling populations for LCS were all private households, excluding institutionalized and homeless people. In both Botswana and Swaziland, a two-stage sampling design was applied. First, enumeration areas (EA) were identified within the national sampling frame based on the most recent census. In both countries, the central statistics office provided the sampling frame. Next, a maximum of 20 household were randomly sampled within each EA to reach the calculated sample size required to produce reliable estimates. An average of 10 of these 20 households had at least one member with a disability.

### Outcome variable

A subset of questions in the LCS were analyzed in order to identify factors associated with AT access. The outcome variable of AT access was captured in the following survey question:

*Which services, if any, are you aware of and have ever needed/received?*

1. *Assistive devices service (*e.g. *Sign language interpreter, wheelchair, hearing/visual aids, Braille* etc.*)*
  A. *Needed service 1 = Yes, 2 = No*
  B. *Received service 1 = Yes, 2 = No*

Cases that responded Yes to 1A. were included for analysis in this study (Table 1). The dichotomous outcome variable is 1B. captures those who received and did not receive AT.

### Explanatory variables

Based on a review of the AT and health services literature from Africa, a number of potential explanatory variables were identified including economic status [19, 20], location (rural vs. urban) [2, 10, 11, 21], education level [20], age, gender, and type of disability [1, 2]. We also elected to include the severity of disability scale to explore the correlation between disability severity and AT access.

In the LCS, disability type was measured by the six questions developed by the Washington Group on Disability Statistics [22], and socioeconomic status was measured in three variables:

- Possession scale - measured ownership of common household items
- Dietary diversity scale - measured types of food intake over the last 2 weeks
- Access to information scale - measured access to common information sources

Descriptions of select explanatory variables are provided in the Additional file 1.

### Statistical analysis

Given the paucity of evidence on AT access specific to Southern Africa, we were not able to develop a specific hypothesis about the order or importance of explanatory variables. Therefore, we applied the statistical (stepwise) logistic regression approach with a bivariate association criterion of *p* <. 20, as recommended by Hosmer and Lemeshow (2000). All potential explanatory variables were analyzed using SPSS bivariate correlation and those that exhibited p <. 20 were included in the final logistic regression models. We then assessed the model’s goodness of fit with the Hosmer & Lemeshow test. SPSS Statistics 24 software was used for all statistical analysis.

## Results

### Characteristics of individuals who needed assistive technology

In Botswana, 574 individuals with disabilities reported needing AT, and 486 of these were 15 years of age or older. Likewise, 496 reported needing AT in Swaziland of which 332 were 15 years or older. Tables 3 and 4 provide the characteristics of individuals with disabilities (age > = 15 years) and the dependent variable of AT access. As shown in Tables 3, 44% (Botswana) and 67% (Swaziland) of the people who needed AT did not receive it. The most common type of disability reported in both countries was difficulty with *Walking/climbing steps* (mobility limitation).

**Table 3:** Characteristics of individuals (15+) who needed AT: Frequencies

	Botswana		Swaziland	
	*N*	%	*N*	%
Total N	486	100.0	332	100.0
AT access (Dependent variable)				
Received AT	272	56.0	104	31.3
Did not receive AT	214	44.0	222	66.9
Missing	0	0	6	1.8
Gender				
Female	216	44.4	180	54.2
Male	263	54.1	152	45.8
Missing	7	1.4	0	0
Locality				
Urban/City	311	64.0	89	26.8
Rural	174	35.8	243	73.2
Missing	1	0.2	0	0
Received a formal primary education				
Received	289	59.5	113	34.0
Did not receive	188	38.7	132	39.8
Missing	0	0	87	26.2
Employed or receiving social grant ^a^				
Yes	217	44.7	74	22.3
No	267	54.9	256	77.1
Missing	2	0.4	2	0.6
Difficulty in: ^a^				
Seeing	169	34.8	73	22.0
Hearing	99	20.4	62	18.7
Walking/climbing steps	324	66.7	216	65.1
Remembering/concentrating	111	22.8	121	36.4
Self-care	214	44.0	119	35.8
Communicating	98	20.2	87	26.2

^a^See Additional file for description of explanatory variables

**Table 4:** Characteristics of individuals (15+) who needed AT: Descriptive statistics

	Botswana			Swaziland		
	*N*	Mean	SD	*N*	Mean	SD
Age	482	48.2	21.4	332	35.2	18.1
Socio-economic status (SES) indicators ^a^						
Possession scale (0–26)	483	8.3	4.9	321	8.4	4.8
Dietary diversity scale (0–12)	468	8.3	2.4	322	8.4	3.0
Access to information scale (0–6)	429	3.3	1.7	319	3.6	1.8
Activity limitations scale (0–18) ^a^	470	4.4	2.7	329	4.1	2.6

^a^See Additional file for description of explanatory variables

### Characteristics of assistive technology acquisition

In both Botswana and Swaziland, the vast majority of recipients of AT reported receiving personal mobility devices (80.1% and 80.8%, respectively). In Botswana, the primary source of AT was the government health service, and AT was usually provided for free. In Swaziland, private suppliers were the most common source identified by recipients of AT (32.7%), whereas the government health service provided AT to only 11.5% (Table 5).

**Table 5:** Individuals who received AT: type and acquisition

	Botswana		Swaziland	
	*N*	%	*N*	%
Total N	272	100.0	104	100.0
Type of AT				
Sensory	19	7.0	6	5.8
Communication	5	1.8	2	1.9
Personal mobility	218	80.1	84	80.8
Other ^a^	9	3.3	1	1.0
Missing (No Type of AT Identified)	32	11.8	16	15.4
Source of AT				
Private	38	14.0	34	32.7
Government health service	123	45.2	12	11.5
Other government service	25	9.2	9	8.7
NGO	27	9.9	11	10.6
Other	34	12.5	19	18.3
Missing	33	12.1	19	18.3
Acquisition of AT ^b^			n.a.	n.a.
Bought it myself	42	15.4	n.a.	n.a.
Bought by someone else	21	7.7	n.a.	n.a.
Given for free	184	67.6	n.a.	n.a.
Missing	33	12.1	n.a.	n.a.

*n.a.* not applicable

^a^Other includes household items, handling products & goods, and personal care & protection products

^b^This survey question was not included in the LCS survey for Swaziland

### Access to assistive technology

Bivariate regressions were conducted to identify variables that were associated with AT access, with a Pearson chi square value criterion of < .20. Table 6 shows positive and negative correlations of factors that met the criterion (*p* < .20) in boldface.

**Table 6:** Bivariate correlations of AT access in Botswana and Swaziland

	Botswana			Swaziland		
	*N*	Pearson’s	Sig. (2-tailed)	*N*	Pearson’s	Sig. (2-tailed)
Gender – Female	475	- 0.065	**0.156**	326	−0.015	0.785
Age	482	−0.050	0.276	326	0.109	**0.050**
Locality – Urban/City	481	0.081	**0.076**	326	0.058	0.295
Received a formal primary education	475	0.157	**0.001**	239	0.173	**0.007**
Employed or receiving grant	480	0.051	0.263	326	0.219	**0.000**
Possession scale	479	0.161	**0.000**	315	−0.067	0.234
Dietary diversity scale	468	0.079	**0.090**	316	−0.045	0.422
Access to information scale	429	0.092	**0.058**	313	−0.051	0.370
Activity limitations scale	466	−0.010	0.826	323	−0.090	**0.108**
Seeing	479	−0.192	**0.000**	324	0.030	0.590
Hearing	473	−0.151	**0.001**	324	−0.082	**0.139**
Walking/climbing steps	477	0.371	**0.000**	323	0.238	**0.000**
Remembering/concentrating	472	−0.156	**0.001**	324	−0.250	**0.000**
Self-care	477	0.046	0.313	324	−0.050	0.372
Communicating	473	−0.101	**0.028**	324	−0.159	**0.004**

Boldface signifies positive and negative correlations of factors that met the criterion (*p* < .20)

For Botswana, four variables were excluded from the model based on the *p* < 0.20 criterion: Age, Employed or receiving grant, Disability severity scale, and Difficulty in self-care. For Swaziland, seven variables were excluded: Gender, Rural or urban, Possession scale, Dietary diversity scale, Access to information scale, Difficulty in seeing, and Difficulty in self-care.

The full models for both Botswana and Swaziland shown in Table 7 are a good fit based on the Hosmer-Lemeshow Test chi-square significance of .227 and .225, respectively (see Additional file 1 for goodness of fit statistics).

**Table 7:** Variables in full models for Botswana and Swaziland

Explanatory variables	Botswana			Swaziland		
	*N*	Exp (B)	[95% CI]	*N*	Exp (B)	[95% CI]
Gender – Female	475	.749	[.464–1.208]	326	n.a.	n.a.
Age	n.a.	n.a.	n.a.	326	1.007	[.991–1.023]
Locality – Urban/City	481	1.293	[.751–2.224]	n.a	n.a.	n.a.
Received a formal primary education	475	1.876^a^	[1.132–3.108]	239	1.683	[.900–3.147]
Employed or receiving grant	n.a.	n.a.	n.a.	326	1.919^a^	[1.029 - 3.578]
Possession scale	479	1.110^b^	[1.030–1.196]	n.a.	n.a.	n.a.
Access to information scale	429	.869	[.715–1.056]	n.a.	n.a.	n.a.
Dietary diversity scale	468	.972	[.866–1.090]	n.a.	n.a.	n.a.
Activity limitations scale	n.a.	n.a.	n.a.	323	1.129	[.898–1.419]
Seeing	479	.507^b^	[.304–.845]	n.a.	n.a.	n.a.
Hearing	473	1.358	[.713–2.589]	324	1.555	[.595–4.060]
Walking/climbing steps	477	6.383^c^	[3.610–11.285]	323	3.183^b^	[1.382 - 7.331]
Remembering/ concentrating	472	.517^a^	[.282–.948]	324	.321^a^	[.132–.782]
Communicating	473	.636	[.335–1.209]	324	.577	[.195–1.705]
Constant		.353	n.a.		.105^c^	n.a.

*n.a.* not applicable

^a^<.05

^b^<.01

^c^<.001

For Botswana, the full model explains the outcome of AT access with 74.2% accuracy in comparison to 57.9% in the null model. Likewise, in Swaziland the full model explains the outcome with 71.3% accuracy in comparison to 64.6% in the null model.

The Botswana model shows that the factor with the strongest association to AT access is disability type, specifically those reporting some level of difficulty in *Walking/climbing steps.* Survey respondents who had a difficulty in *Walking/climbing* steps were 6.4 times more likely to have access to AT than those who did not report this type of difficulty. However, those who reported difficulty in *Seeing* and *Remembering/concentrating* were over 50% less likely to access AT. In addition, those who completed formal primary education were nearly twice as likely to have access to AT than those who did not complete primary education. The possession scale was also significantly associated with AT access though shows little change in likelihood. For every unit increase (O-18 possessions), respondents were 1.11 times more likely to have AT access. When controlling for other variables, gender, location, and socioeconomic status variables (i.e., access to information, dietary diversity), and some disability types (i.e., hearing, communicating) were not significantly associated with AT access.

Similar to Botswana, the Swaziland model shows that the factor with the strongest association with AT access was disability type (i.e., difficulty in *Walking/climbing steps).* Survey respondents who had a difficulty in *Walking/climbing* steps were 3.2 times more likely to have access to AT than those who did not report this type of difficulty. However, those who reported difficulty in *Remembering/concentrating* were 58% less likely to access AT. The only other significant explanatory variables of AT access in Swaziland was *Employment/receiving grant.* Those who were employed, receiving disability or other grant were nearly twice as likely to access AT as those who were not employed or receiving grant. Unlike Botswana, education status was not found to be significantly associated with AT access in Swaziland when controlling for other variables.

**Fig. 1:**
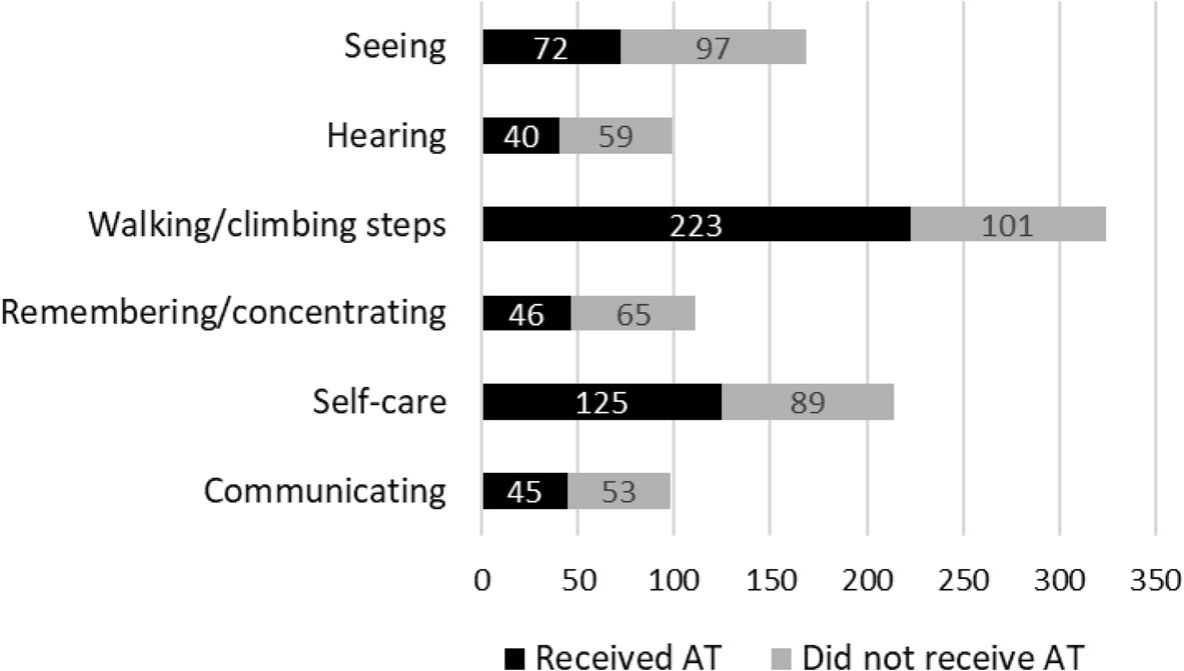
Access to AT by disability type in Botswana

**Fig. 2:**
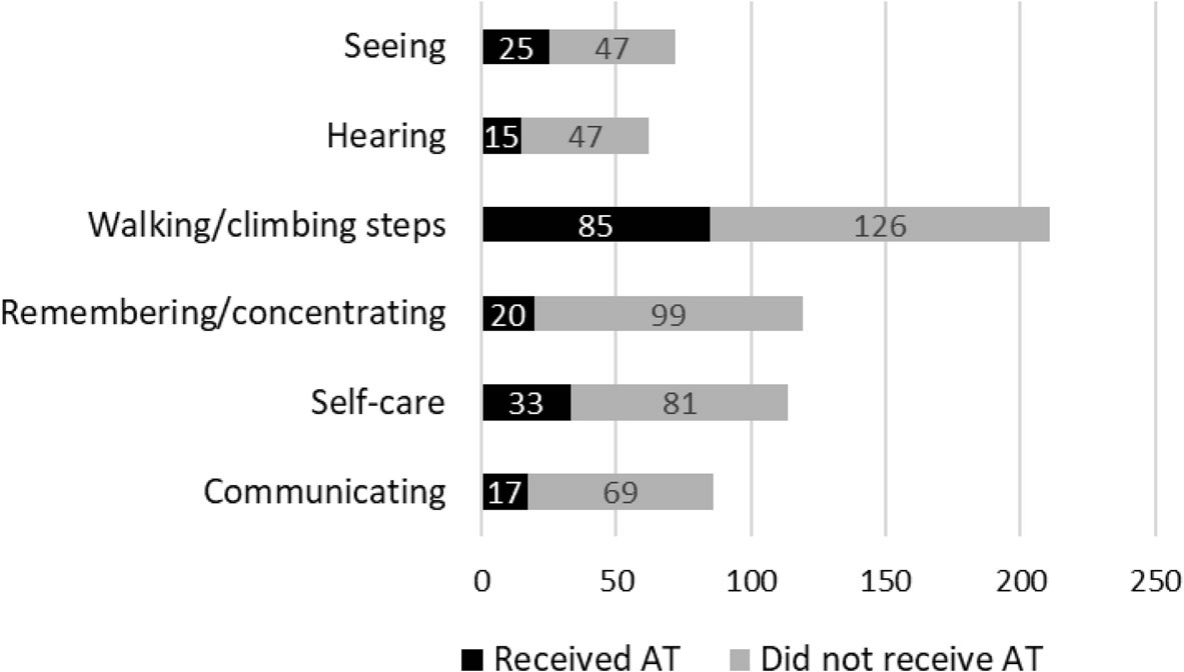
Access to AT by disability type in Swaziland

While our statistical model shows that disability type (i.e., mobility restrictions) is the most important explanatory variables of AT access in both countries, there is a large unmet need for AT across all disability types in both countries, including those who report difficulties with *Walking/climbing steps* (Figs. 1 and 2). Mobility is also the category with the highest total number of individuals without access to AT in both countries (Botswana = 101, Swaziland = 126).

**Fig. 3:**
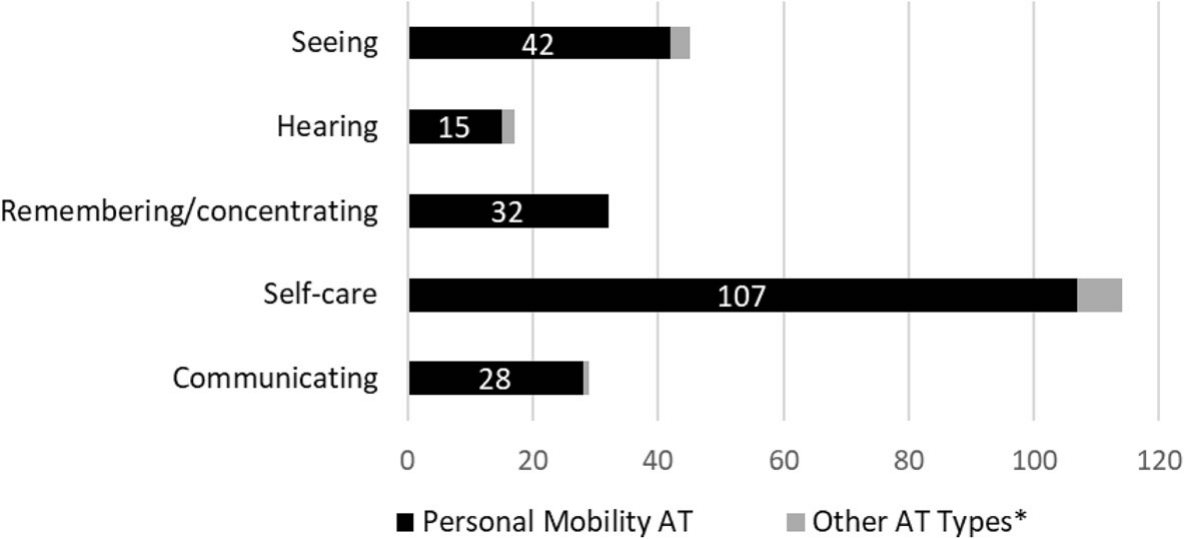
Disability by AT type: Recipients of AT with mobility limitations in Botswana. *Non-personal mobility AT such as sensory, communication, household items, handling products & goods, and personal care & protection products

**Fig. 4:**
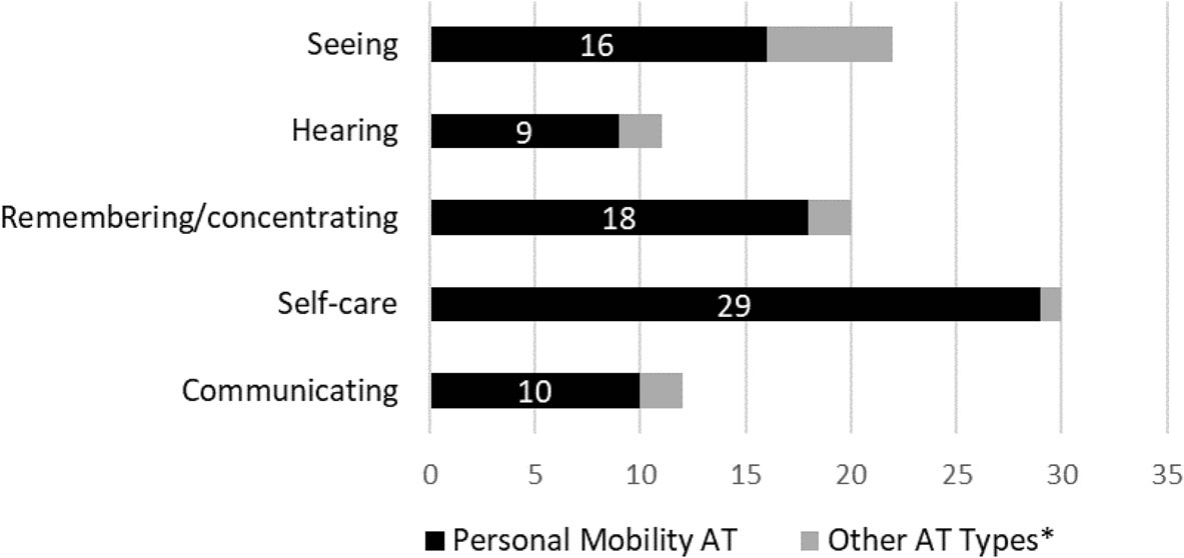
Disability by AT type: Recipients of AT with mobility limitations in Swaziland. *Non-personal mobility AT such as sensory, communication, household items, handling products & goods, and personal care & protection products

If we examine the subgroup of people with mobility limitations who received AT (Botswana = 223; Swaziland = 85), we see that many reported having other non-mobility types of disabilities, and that personal mobility AT dominated across all these other disability types (Figs. 3 and 4). This shows that the type of AT received often does not correspond with non-mobility types of disability, further demonstrating the dominance of personal mobility devices in the AT sector.

## Discussion

These statistical results serve to identify factors that are associated with AT access in Botswana and Swaziland. The most notable finding in both countries is that people with mobility restrictions are most likely to access AT, irrespective of all other sociodemographic factors such as age, gender, socioeconomic status, education level or disability severity. While there is a large unmet need for all types of AT in the both countries, the current coverage levels of AT are not proportional to prevalence of disability types (mobility, seeing, communication, etc.). For example, in Botswana 60% of those with hearing difficulties and 31% of those with mobility difficulties did not have access to AT. In addition, for recipients of AT who had mobility limitations in combination with other types of disabilities, the type of AT received was heavily dominated by personal mobility AT regardless of what may have been the primary disability type (e.g., Seeing).

An explanation for higher AT access among those with mobility difficulties is that mobility is the most prevalent type of disability in both countries (Table 3) and in other Southern African countries [23, 24], and has thus logically has received the most attention and resources within the regional AT sector. This finding also reflects a number of global AT trends. First, there is a greater awareness of and access to mobility devices (wheelchairs, crutches, prosthetics) in Southern Africa and other LMICs than other categories of AT such as for hearing, vision, communication, and cognition [2, 25]. Second, international agencies, NGOs and charity organizations have devoted greater financial resources to mobility devices (i.e., wheelchairs) than other types of AT [26, 27]. Finally, evidence reviews on AT research in LMICs show that the research community has also prioritized mobility studies over other AT categories [7, 28, 29]. This focus on mobility devices is starting to expand with increased awareness about the needs and rights of broader populations who benefit from AT such as people who are aging [30], people with intellectual, development or mental health impairments [31], and people with rare disabilities (e.g., albinism) [32].

The emphasis of the AT sector on mobility devices is most pronounced in Botswana where a person with a mobility impairment is over six times more likely to have access to AT than a person with a non-mobility type of disability. Given that the government health service was the source of AT for over 45% of AT recipients, it is likely that national AT budgets are devoted to a narrow range of mobility-related AT (i.e., wheelchairs, crutches, walkers). Expanding the range of AT covered by the relevant ministries within the public sector (i.e., health, social development and education), and provided by development partners is one of the aims of the WHO GATE initiative [33]. To achieve this aim GATE launched the Priority Assistive Product List (APL) [34], a list of 50 essential assistive products that, if provided, propose to address the greatest unmet AT needs globally.

Another key finding of this study is that both the unmet AT need (percentage that did not receive AT) and explanatory factors of AT access vary by country. A higher percentage of people received AT in Botswana (56.0%) than Swaziland (31.3%). This is not surprising given the lower development indicators for Swaziland as shown in Table 2. There were only two common explanatory variables in both Botswana and Swaziland models; 1) those with difficulty in *Walking/climbing steps were 6.4 and 3.2 times more likely to access AT* than those without mobility limitations, and those with difficulty in *Remembering/concentrating* were 50% and 58% less likely to access AT than those without this limitation. In Botswana, difficulty in *Seeing* was also negatively associated with AT access, and completing a formal primary education and having more possessions were positively associated. In Swaziland, *Employment/receiving grant* was the only other significant explanatory variable of AT access. This is consistent with previous finding that there was substantial variation in AT access between countries, and that mobility devices are most commonly available [2]. The differences in factors that explain AT access between the two countries may indicate variations in the procurement and distribution mechanisms within each country. The results from this study and other literature (e.g. Visagie et al. 2017) indicate a more developed public sector for AT in Botswana than in Swaziland. Access to AT in Swaziland is most commonly achieved through purchasing on the private market or being given devices through charity/donation based providers, while in Botswana one needs to interact with the public system to obtain AT. This may help explain the importance of education in Botswana, specifically that higher education correlates with higher public sector access, because those who are accessing public education are more likely to be informed about and able to access other public services (i.e., health) than those who have not received a formal primary education. Likewise, the importance of employment or receiving a grant in Swaziland could reflect the dominance of the private sector as one has to pay out of pocket to receive AT.

It is important to note that the significance of mobility impairments within both country logistic regression models does not imply that other sociodemographic characteristics such as gender, location and age do not affect access to AT, as all these factors independently have been shown to be associated with AT access in bivariate correlations (Table 6). In the above regression models, the impact of other factors are largely mediated by mobility impairments.

### Limitations

The primary limitations of this study are that both datasets are not recent (i.e., data collection was conducted in 2012–14 in Botswana and 2009–10 in Swaziland), and the survey question for the outcome variable did not specify a timeframe so respondents could be referring to AT access at any timeframe in the past. Other limitations include possible missing explanatory variables (e.g., access to transportation), self-reported data could be subject to recall and self-report bias, and low levels of awareness about less commonly available type of AT such as AT for communication or cognition resulted in underreporting. In addition, datasets from only 2 of the 15 countries in Southern Africa may not be representative of the region as a whole. Despite these limitations, this study provides evidence that the AT sector in Southern Africa is heavily dominated by mobility devices, so much so that none of the tested sociodemographic characteristic (e.g., age, gender, locality, education level) explain AT access as strongly as type of disability.

## Conclusion

Governments and other stakeholders in the AT sector in Southern Africa must prioritize AT to address the large unmet demand across all types of AT, and in order to meet obligations of the CRPD and the Sustainable Development Goals. These findings also provide support for expanding the range of AT products provided so that people with hearing, seeing, self-care, communication and cognition difficulties have equal access to AT as those with mobility impairments. A step toward achieving these aims is to inventory AT product types that are commonly covered through the public sector in each country, and identify common gaps (e.g., daily living aids). The Priority Assistive Products List [34] established by WHO’s GATE can serve as an inventory taking tool, that can be adapted to match the unique AT needs and strengths within each country.

Advancing the AT sector within Southern Africa will require a significant investment in resources by the international and global health communities, along with local governments - both to develop a comprehensive understanding of the bottlenecks in AT procurement and service delivery systems, and test and apply system-level inventions.

## Additional file

Additional file 1: **Table S1.** Description of explanatory variables. **Table S2.** Botswana: Bivariate Logistics Regression – Goodness of fit. **Table S3.** Swaziland: Bivariate Logistics Regression – Goodness of fit. (DOCX 35 kb)

## Abbreviations

APL: Priority Assistive Product List
AT: Assistive technology
CRPD: United Nations Convention on the Rights of Persons with Disabilities
EA: Enumeration areas
GATE: Global Cooperation on Assistive Health Technology
LCS: Living Conditions Studies
