# Supplementary material for: Access to assistive technology in two Southern African countries
Source: BMC Health Serv Res. 2018 Oct 19;18:792. doi: 10.1186/s12913-018-3605-9 (PMC6194741; doi:10.1186/s12913-018-3605-9)
Supplement: Supplementary file 1 — Table S1. Description of explanatory variables. Table S2. Botswana: Bivariate Logistics Regression – Goodness of fit. Table S3. Swaziland: Bivariate Logistics Regression – Goodness of fit. (DOCX 35 kb) [file 12913_2018_3605_MOESM1_ESM.docx]

## Additional file 1

**Table S1 Description of explanatory variables**

| **Explanatory variables** | | **Survey items** | **Response options** |
| --- | --- | --- | --- |
| Employed or receiving social grant | | Are you currently working? (includes casual labourers, part-time work and those who are self-employed). | Recoded:  Yes = 1 (respondents who reported that they are ‘currently working’ or are receiving social security grant, etc.)  No = 0 (not working and not receiving grant). |
|  |  | Are you currently receiving social security, a disability grant or any other form of pension/grant? |  |
| Activity limitation questions: | Seeing | Do you have difficulty seeing, even if wearing glasses? | No difficulty = 0  Some = 1  A lot = 2  Unable = 3  Recoded:  Yes = 1 (respondents who had any level of difficulty - Some, A lot, or Unable);  No = 0 (No difficulty). |
|  | Hearing | Do you have difficulty hearing, even if using a hearing aid? |  |
|  | Walking/ climbing steps | Do you have difficulty walking or climbing steps? |  |
|  | Remembering/ concentrating | Do you have difficulty remembering or concentrating? |  |
|  | Self-care | Do you have difficulty with self-care such as washing all over or dressing? |  |
|  | Communicating | Using your usual (customary) language, do you have difficulty communicating for example understanding or being understood? |  |
| Activity limitations scale (0-18) | | Calculated: Numeric responses (0-3) to the six activity limitation questions were added together to form an Activity Limitations Scale. Values ranged from 0 to 18. | |
| Possession scale (0-26) | | Does your household have any of the following?   1. Cell phone 2. Bed(s) 3. Tables & chairs 4. Iron 5. Stove (gas/electric) 6. Electricity 7. TV 8. Radio 9. Refrigerator 10. Satellite dish 11. DVD/VHS 12. Livestock 13. Car 14. Microwave oven 15. Fan 16. Hi-Fi 17. Telephone (land line) 18. Heater 19. Computer 20. Bicycle 21. Stove (paraffin) 22. Washing machine 23. Solar energy 24. Air conditioner 25. Electrical generator 26. Motorcycle | Yes = 1  No = 0 |
| Dietary diversity scale (0-12) | | Now I would like to ask you about the types of foods that you or anyone else in your household prepared and ate in the past TWO weeks during the day and night (food purchased and eaten outside of the home is not included).   1. Cereals 2. Roots and tubes (veg) 3. Leaf vegetables 4. Fruits 5. Meat, poultry, offal 6. Eggs 7. Fish and seafood 8. Pulses/legumes/nuts 9. Milk and milk products 10. Oil/fats 11. Sugar/honey 12. Condiments and any other foods | Yes = 1  No = 0 |
| Access to information scale (0-6) | | How available are the following services to your household?   1. Telephone/cell 2. Radio 3. Television 4. Internet 5. Newspaper 6. Library | Yes = 1 (Own/use regularly or Have access to)  No = 0 (Have no use for, Have no access to) |

**Table S2. Botswana: Bivariate Logistics Regression – Goodness of fit**

| **Model Summary** | | **Hosmer and Lemeshow Test** | |
| --- | --- | --- | --- |
| -2 Log likelihood | 427.767 | Chi-square | 10.574 |
| Cox & Snell R Square | .237 | df | 8 |
| Nagelkerke R Square | .318 | Sig. | .227 |

**Table S3. Swaziland: Bivariate Logistics Regression – Goodness of fit**

| **Model Summary** | | **Hosmer and Lemeshow Test** | |
| --- | --- | --- | --- |
| -2 Log likelihood | 265.438 | Chi-square | 10.614 |
| Cox & Snell R Square | .165 | df | 8 |
| Nagelkerke R Square | .227 | Sig. | .225 |
